# Comparative Analyses of Gut Microbiomes in *Hycleus cichorii* (Coleoptera: Meloidae) Adults Reveal Their Distinct Microbes, Microbial Diversity and Composition Associated to Food

**DOI:** 10.1002/ece3.70948

**Published:** 2025-01-30

**Authors:** Chunyan Yi, Li Gao, Cuicui Zhang, Yanping Wang, Xu Liu, Yongli Yang, Song Chen, Chao Du

**Affiliations:** ^1^ Key Laboratory of Integrated Pest Management of Southwest Crops, Institute of Plant Protection Sichuan Academy of Agricultural Sciences Chengdu China; ^2^ Baotou Teachers College Baotou China; ^3^ Panzhihua Academy of Agriculture and Forestry Sciences Panzhihua China

**Keywords:** feeding habits, food, full‐length 16S rDNA, gut microbiota

## Abstract

Gut microbiota crucially affects metabolism and health. *Hycleus cichorii* Linnaeus has been listed as a medicinal insect in the Pharmacopeia of the People's Republic of China due to the presence of cantharidin, which has a curative effect on many cancers and skin diseases. In order to analyze the effects of dietary habits and gender on the diversity and composition of gut microbiota in *H. cichorii* and provide a basis for an artificial diet, in this study, the full‐length 16S rRNA sequencing technology was used to analyze the gut microbiota of 35 *H. cichorii* adults, including wild female adults (WFA), wild male adults (WMA), female adults fed with luffa flowers (LFA), male adults fed with luffa flowers (LMA), female adults fed with artificial diet (AFA), and male adults fed with artificial diet (AMA). The results displayed that the major bacterial phyla present in the gut microbiota of the *H. cichorii* were Firmicutes, Proteobacteria, Bacteroidetes, Fusobacteria, Teneriicutes, and Actinobacteria. The major bacterial genera were *Lactococcus*, *Lactobacillus*, *Enterococcus*, *Ralstonia*, *Sebaldella*, *Dysgonomonas*, *Spiroplasma*, *Weissella*, *Klebsiella*, and *Serratia*. Food habits had a significant effect on the diversity and composition of gut microbiota in *H. cichorii*, whereas gender did not exhibit a remarkable impact on the diversity and composition of gut microbiota. The artificially fed group of *H. cichorii* had more beneficial microorganisms in the intestine and higher food utilization efficiency. These results provide a basis for subsequent examination of gut microbiota in *H. cichorii* or other Coleoptera insects, as well as the artificial rearing of blister beetles.

## Graphical abstract

The study analyzed the gut microbial diversity and composition of H. cichorii with different dietary habits and genders. The results showed that an elevated richness in food composition corresponds to a higher diversity of gut microbial species in the host. It suggests that food plays a significant role in the composition of the gut microbial community, which may consequently affect the nutritional metabolism and growth of the host.

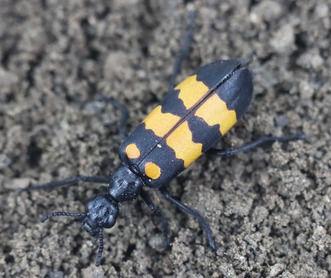

**Abbreviations**
AFR: artificial fed female adults
AFS: female adults fed with luffa flowers
AFY: wild female adults
AMR: artificial fed male adults
AMS: male adults fed with luffa flowers
AMY: wild male adults
AR: adults fed with artificial diet
AS: adults fed with luffa flowers
AY: wild female adults
CTAB: Hexadecyl trimethyl ammonium Bromide
LDA: Linear discriminant analysis
LEfSe: Linear discriminant analysis (LDA) effffect size
NMDS: Non‐metric multidimensional scaling
OTUs: operational taxonomic units
PCR: polymerase chain reaction

## 1 Introduction

Studies have shown that symbiotic microorganisms exist in the gut of insects (Baumann et al. 1995; Tan et al. 2005; Du, Li, and Liu 2020). With the development of high‐throughput sequencing technology, an increasing number of insect gut microorganisms have attracted attention (Yuan et al. 2021). These symbiotic microorganisms play important roles in various aspects of insect growth, development, food decomposition, immunity, and other functions (Cazemier et al. 1997). As an important component of the insect digestive system, the gut plays a significant role in the selection of insect feeding habits and the degradation and metabolism of food by symbiotic bacteria (Zchori‐Fein and Brown 2002; Chandler, Wilkinson, and Douglas 2008; Xu et al. 2009; Sharon et al. 2010; Provorov and Onishchuk 2018). Food composition also has a significant impact on the gut microbiota of insects (Yang et al. 2020). The study conducted by Zheng (2023) showed that the dietary habits of insects in the Ensifera were closely related to the composition of gut bacterial communities. Insects of different species with the same dietary habits had similar gut bacterial communities, whereas significant differences were noted in the composition and function of gut microbial communities in the same species of insects with different dietary habits.

**FIGURE 1:**
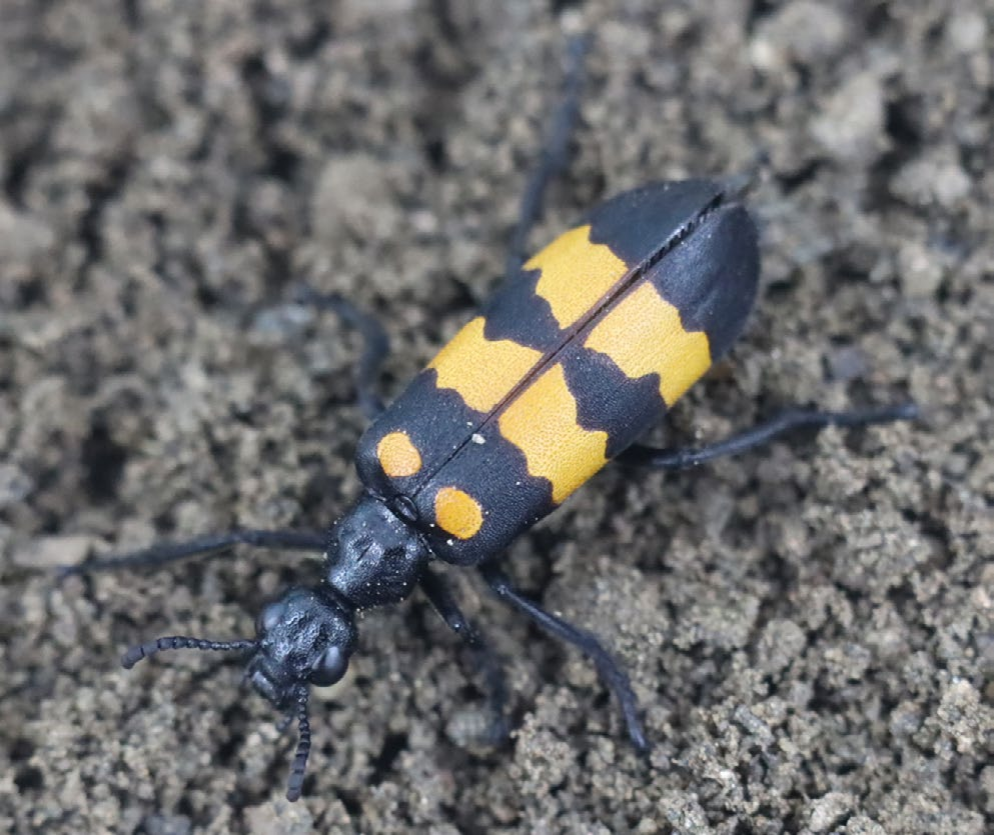
Organism photograph (*Hycleus cichorii*).

*Hycleus cichorii* Linnaeus is commonly known as the “yellow and black small blister beetle” (Figure 1). Blister beetle is a general term for the Meloidae family of insects in the Coleoptera order. It was included as a medicinal insect in the Pharmacopeia of the People's Republic of China (2005) due to the presence of the medicinal substance cantharidin. The utilization of cantharidin is expanding across various applications, leading to a growing demand for cantharidin resources (Ma et al. 2024). However, due to unrestrained and excessive capture of the *H. cichorii*, wild cantharidin resources have gradually decreased. The limited availability of meloid resources is hindering the progress and utilization of cantharidin. Consequently, the artificial rearing of blister beetles has become an inevitable trend to overcome this bottleneck. Several studies have been undertaken to explore the artificial breeding of *H. cichorii*, focusing on determining the optimal conditions for growth, including temperature, humidity, light, and feeding density (Zhang and Hu 1988; Hu, Zhang, and Wei 1993). Its larvae feed on locust eggs (or parasitic bee nests, feeding on the eggs, larvae, and nectar of ground wasps), whereas the growth of adults relies on host plants, such as loofah flowers, rendering artificial raising difficult. Some scholars have studied the artificial feed formula for adult and larval *H. cichorii* but cannot fully mediate artificial feeding (Wang 2008). A thorough understanding of the gut microbiota and diversity of the beetle can provide a theoretical basis for exploring the symbiotic relevance between gut microbiota and host, as well as the mechanism of host‐microbiota interactions.

Different types of food can alter the host's gut microbiota, thereby adapting to the processes of nutrient metabolism, growth, and development in the organism (Xiao et al. 2021). However, no relevant reports on the gut microbiota of *H. cichorii* have been published. The current study analyzed and compared the symbiotic bacteria diversity in the gut of *H. cichorii* adults feeding on different foods using high‐throughput sequencing technology to determine the impact of dietary habits and gender on the composition and diversity of gut microbiota.

## 2 Materials and Methods

### 2.1 Samples Information

**FIGURE 2:**
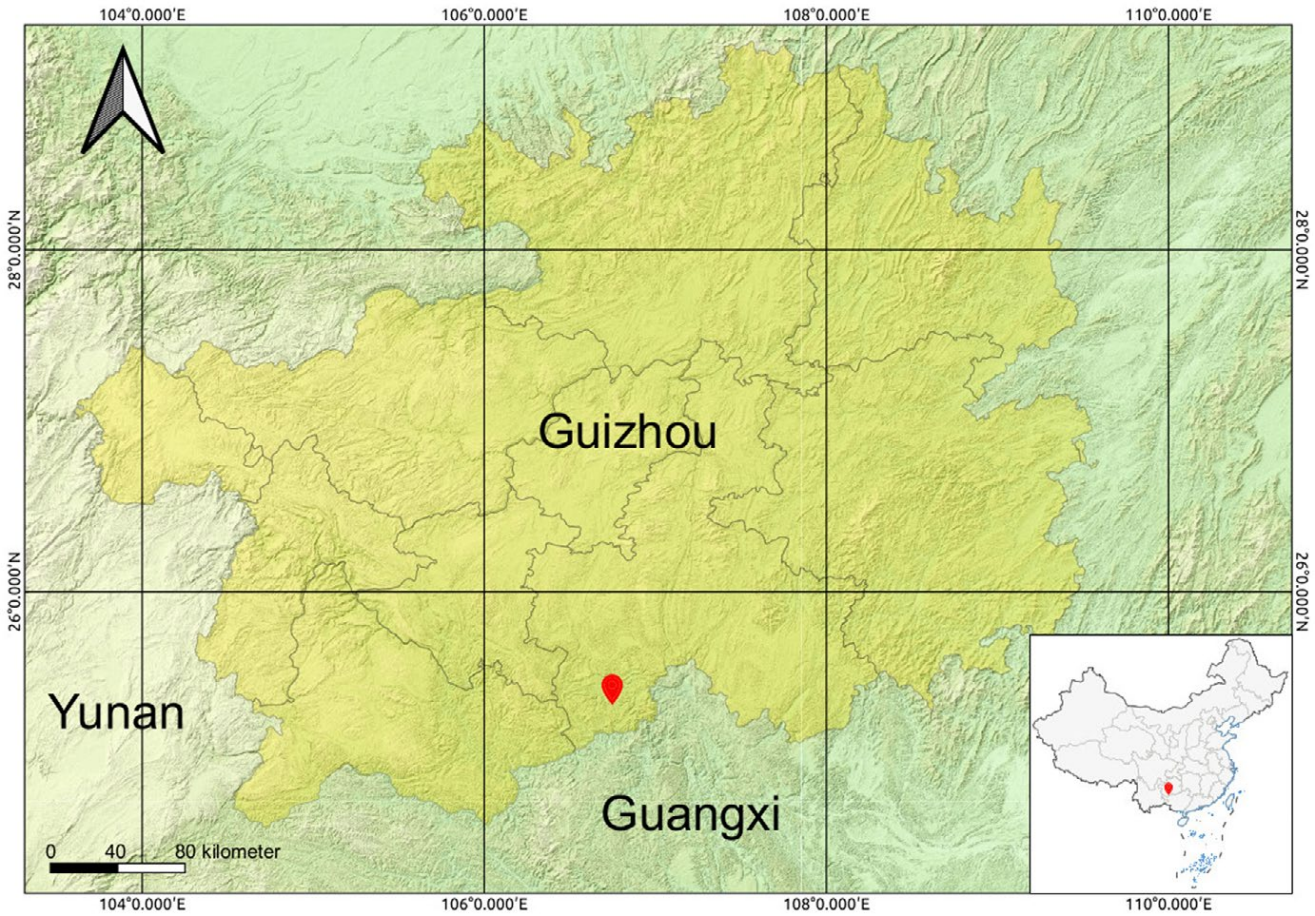
The sampling site map.

35 *H. cichorii* adults including 6 wild females (WFA) and 6 males (WMA), 6 luffa flower‐fed females (LFA) and 6 males (LMA), and 6 artificial fed females (AFA) and 5 males (AMA) were used as samples. Wild *H. cichorii* adults are mainly distributed in the southern region of China at an altitude of less than 2000 m in the hilly mountainous areas with vegetative cover. Its primary dietary sources are the flowers of leguminous and cucurbitaceous plants. The *H. cichorii* adults were collected from Luodian County, Guizhou Province, China (Figure 2). The adults were placed into an insect‐rearing cage and immediately brought back to the laboratory and identified according to their morphological features. The WFA and WMA were sampled immediately for gut extractions. Other wild beetles were randomly grouped and raised in two plastic boxes with a ventilation cover and soil on the bottom. The beetles in two groups were separately fed with an excess of luffa flowers or artificial diet for more than 7 days for the next sampling preparations. The LFA, LMA, AFA, and AMA were sampled from the two groups that were separately fed luffa flowers and artificial feed. The rearing condition was a temperature of 29°C ± 1°C and a relative humidity of 60% ± 5%. Further details as described in the study by Fu, Liu, and Du (2023). The components of the artificial diet were 8.8 g of soybean powder, 6 g of yeast powder, 5 g of dextrose, 1.5 g of milk powder, 0.3 g of sorbic acid, 0.3 g of Vitamin C, 0.7 g of maize pollen, 0.7 mL of honey, 1 g of agar, 0.7 g of royal jelly, and 75 g of water. The sterile water and agar were boiled and then mixed with the other ingredients, which were previously sterilized for 30 min at 121°C.

### 2.2 Sample Anatomy and Intestinal Collection

The collected samples were transferred to a new centrifuge tube, followed by starving in a feeding environment for 24 h to achieve a relatively stable level of gut microbiota. The starved *H. cichorii* were rinsed with sterile water once, soaked in 75% ethanol for 2 min, and rinsed again with sterile water to remove residual ethanol. Pointed forceps were used to unfold the abdomen under a stereomicroscope. Subsequently, the intestines were harvested, placed in sterile centrifuge tubes, frozen in liquid nitrogen, and stored in a refrigerator at −80°C until further analysis.

### 2.3 Full‐Length 16S rDNA Sequencing of Gut Symbiotic Microbiota

The genomic DNA of intestinal bacteria was extracted by the CTAB method. The DNA concentration was determined, and the integrity of DNA was detected by agarose gel electrophoresis. The bacterial 16S rRNA gene full‐length was amplified using the primers 27F (AGAGTTTGATCCTGGCTCAG) and 1492R (GNTACCTTGTTACGACTT) (Zhang et al. 2024). The PCR amplicons were assayed by 2% agarose gel electrophoresis. The same amount of samples was mixed according to the concentration of PCR products. After fully mixing, 2% agarose gel electrophoresis was used to detect the PCR products. The products were recovered, and then the sequencing connector was attached to both ends of the amplified DNA fragment using a DNA adhesive enzyme. The selected DNA fragment was purified using AMPure PB magnetic beads, and the SMRT Bell library was constructed. After the purified fragments were dissolved in the buffer, BluePipin fragments were used to screen specific‐sized fragments, and the DNA fragments were purified using AMPure PB magnetic beads. The constructed library was quantified using Qubit concentration, and the inserted fragment size was detected using Agilent 2100, followed by sequencing using the PacBio platform.

### 2.4 Bioinformatics Analysis

PacBio offline data was exported to a BAM format file. Lima software was used to distinguish the data of each sample based on barcode sequences, and the sequences of all samples were saved in Bam format. Then, the sequences were corrected using CCS (SMRT Link v7.0) with a correction parameter of CCS = 3 and a minimum accuracy of 0.99. Sequences with lengths less than 1340 and longest sequence lengths greater than 1640 were removed and saved in fastq and fasta. Subsequently, SSR filtration was performed, and primers were removed using cutadapt to filter out sequences containing consecutive identical base numbers > 8. A total of 440,074 reads were obtained after the aforementioned processing and considered the valid data (clean reads).

All clean reads of all samples were clustered using Uparse software (Edgar 2013) with a default consistency of 97% (identity) to cluster the sequences into OTUs (Operational Taxonomic Units). The sequences with the highest occurrence frequency in OTUs were simultaneously screened as the representative sequences of OTUs. Subsequently, species annotation analysis was performed to examine the representative sequence of OTUs using the Mothur method (Schloss et al. 2009) and the SSUrRNA database in SILVA (http://www.arb‐silva.de/) with a threshold of 0.8–1, obtaining taxonomic information, and statistically analyzing the community composition of each sample at various taxonomic levels: kingdom, phylum, class, order, family, genus, and species. Using MUSCLE (Edgar 2004) software, rapid multi‐sequence alignment was performed to harvest the systematic relationships of all OTUs representing sequences. Finally, the data of each sample was homogenized, with the least amount of data in the sample as the standard for homogenization. Subsequent alpha diversity analysis and beta diversity analysis were based on the homogenized data.

The data of each sample were normalized based on the minimum amount of data. Furthermore, the Chao1 and ACE indices were calculated using the Qiime software (Caporaso et al. 2010). The differences in the alpha diversity indices between different groups were examined using a *t*‐test.

Weighted and unweighted Unifrac distances were calculated using the Qiime software (Caporaso et al. 2010), and the NMDS diagram was drawn using the Vegan software package of R software (Version 2.15.3). Linear discriminant analysis (LDA) effect size (LEfSe) was applied to analyze bacteria with significant differences between groups (LDA > 4.0) using the LEfSe software (Segata et al. 2011). The specific method is described as follows: First, the non‐parametric factor Kruskal‐Wallis test was used to detect species with significant differences in abundance between different groups. Then, the Wilcoxon rank sum test was used to check whether all subspecies in significantly different species tended to cluster at the same classification level. Finally, linear discriminant analysis (LDA) was used to evaluate the data and identify the species with the most significant differences (LDA > 4.0, *p* < 0.05).

## 3 Results

### 3.1 Alpha Diversity Analysis

**FIGURE 3:**
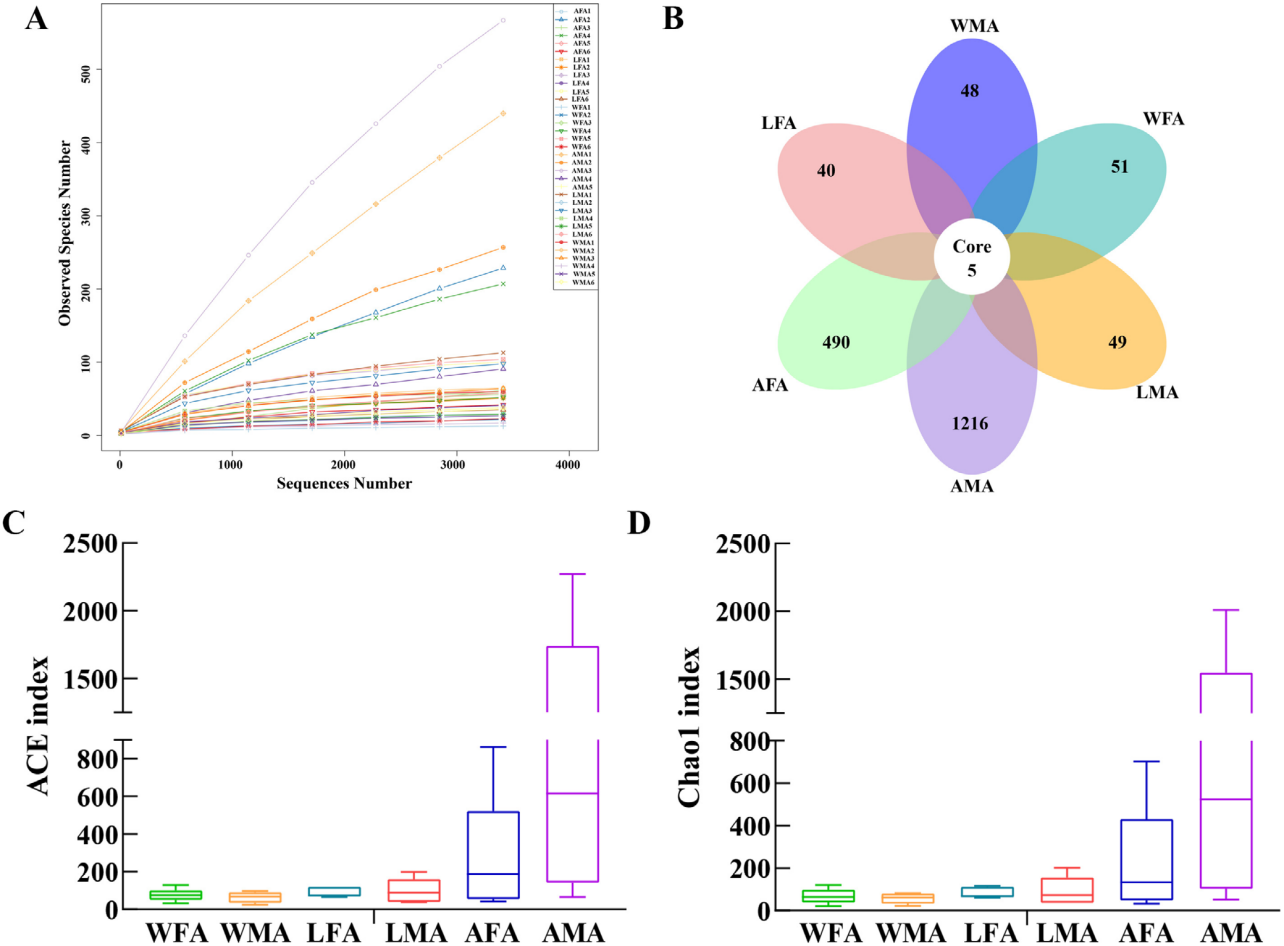
α Diversity analysis of the bacteria community in *H. cichorii* in the six groups. (A) Rarefaction curves of the bacterial community, *X*‐axis: Observed species number in each sample, *Y*‐axis: Sequence number in each sample; (B) Venn diagram of the OTU levels of the six groups; (C) ACE indices of the bacterial community of the six groups, *X*‐axis: Groups, *Y*‐axis; ACE index; (D) Chao1 diversity of the bacterial community of the six groups, *X*‐axis: Groups, *Y*‐axis: Chao1 index. AFA, artificially fed female adults; AMA, artificially fed male adults; LFA, female adults fed with luffa flowers; LMA, male adults fed with luffa flowers; WFA, wild female adults; WMA, wild male adults.

At a sequence similarity level of 97%, a total of 3284 OTUs were acquired and annotated into 18 phyla, 27 classes, 58 orders, 94 families, and 189 genera. The rarefaction curve achieved a plateau (Figure 3A), indicating that as the sequencing depth increased, the microbial diversity in these samples no longer increased. The quantity of sequencing data employed in the study was judiciously selected, allowing for a representative portrayal of the prevailing microbial composition within the sample. In terms of OTUs, there were 5 core OTUs, 51 WFA‐specific OTUs, 48 WMA‐specific OTUs, 40 LFA‐specific OTUs, 49 LMA‐specific OTUs, 490 AFA‐specific OTUs, and 1216 AMA‐specific OTUs were noted in six groups of samples (Figure 3B). At the same sequencing depth, ACE and Chao1 indices were used to analyze the ɑ diversity in these samples. As shown in Figure 3C,D, no remarkable differences in the ɑ diversity indices were observed among six groups of samples (*p* > 0.05).

### 3.2 Composition Analysis of Gut Microbiota in Different Groups of Samples

**FIGURE 4:**
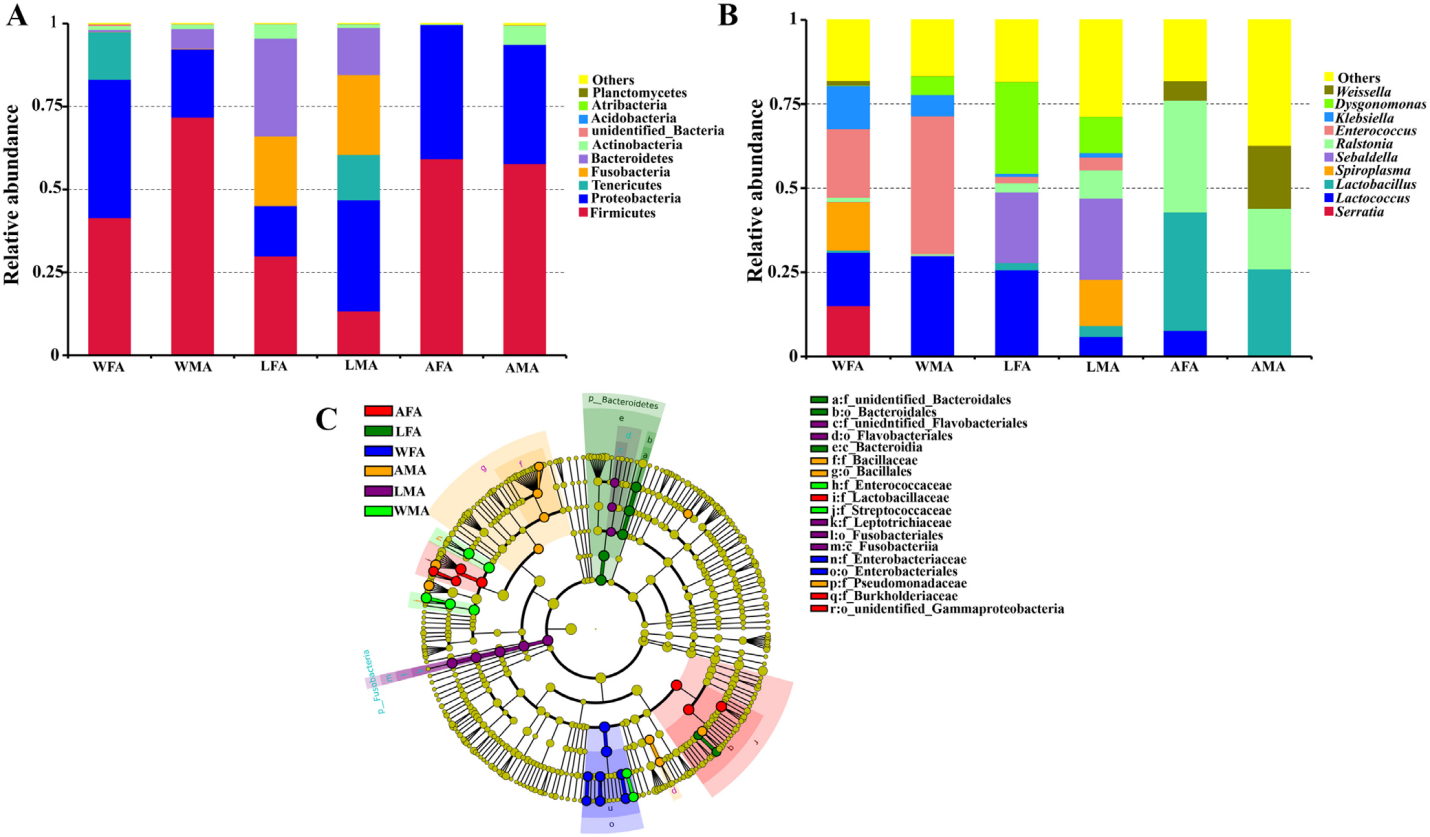
Composition analysis of gut bacteria in *H. cichorii* of the six groups. (A) Top 10 bacterial phylum with relative abundance, *X*‐axis: Groups, *Y*‐axis: Relative abundance of the bacteria; (B) Top 10 bacterial genera with relative abundance, *X*‐axis: Groups, *Y*‐axis: Relative abundance of the bacteria; (C) Linear discriminant analysis effect size (LEfSe) analysis. The figure exhibited microbial counts with significant differences between the six groups. (LDA > 4.0, *p* < 0.05). AFA, artificially fed female adults; AMA, artificially fed male adults; LFA, female adults fed with luffa flowers; LMA, male adults fed with luffa flowers; WFA, wild female adults; WMA, wild male adults.

Statistical analysis was conducted to examine bacterial phyla and bacterial genera with an abundance of more than 1% in each group of samples (Figure 4). At the phyla level, Firmicutes (45.63%), Proteobacteria (31.18%), Bacteroidetes (8.37%), Fusobacteria (7.55%), Teneris (4.67%), and Actinobacteria (2.32%) had an average relative abundance of more than 1% (Figure 4A). At the genus level, the most abundant bacterial genera were *Lactococcus* (14.18%), *Lactobacillus* (11.22%), *Enterococcus* (11.19%), *Ralstonia* (10.67%), *Sebaldella* (7.56%), *Dysgonomonas* (7.27%), *Spiroplasma* (4.67%), *Weissella* (4.36%), *Klebsiella* (3.56%), and *Serratia* (2.54%). The remaining genera accounted for 22.80% (Figure 4B). LEfSe analysis was conducted to analyze the differences in bacterial composition between different groups of samples (Figure 4C). No iconic phylum of bacteria was noted in the WFA, WMA, AFA, and AMA groups. The iconic phylum was Bacteroidetes in the LFA group and Fusobacteria in the LMA group (LDA > 4, *p* < 0.05). At the genus level, the iconic bacterial genera were *Serratia*, *Klebsiella*, and *Citrobacter* in the WFA group, *Enterococcus, Lactococcus*, and *Unidentified_ Enterobacteriaceae* in the WMA group, *Dysgonomonas and Comamonas* in the LFA group, *Sebaldella* and *Empedobacter* in the LMA group, *Ralstonia, Lactobacillus*, and *Pediococcus* in the AFA group, and *Cupriavidus, Bacillus, Glutamicibacter, and Pseudomonas* in the AMA group (LDA > 4, *p* < 0.05) (Figure 4C).

### 3.3 Beta Diversity Analysis

**FIGURE 5:**
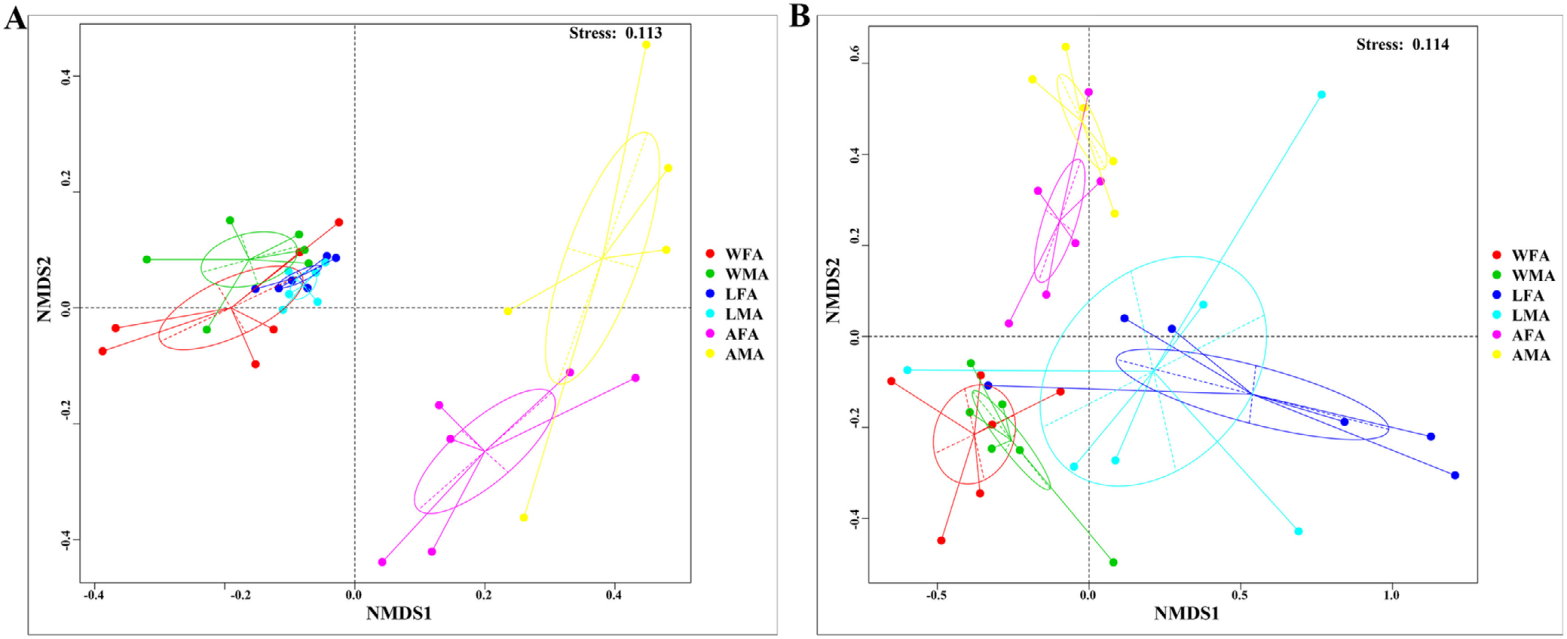
Beta analysis of the bacterial community in *H. cichorii* of the six groups. (A) NMDS analysis based on unweighted UniFrac distance; (B) NMDS analysis based on weighted UniFrac distance. AFA, artificially fed female adults; AMA, artificially fed male adults; LFA, female adults fed with luffa flowers; LMA, male adults fed with luffa flowers; WFA, wild female adults; WMA, wild male adults.

Beta diversity analysis was performed by unweighted UniFrac NMDS and weighted UniFrac NMDS. The unweighted UniFrac NMDS analysis shows that different samples exhibited clustering trends, with AFA and AMA being far away from the other four groups of samples, indicating significant differences in microbial composition between these two groups of samples and the other groups of samples (Figure 5A). The weighted UniFrac‐NMDS analysis showed that female and male samples with the same feeding habits showed a clustering trend (such as WFA and WMA, LFA and LMA, and AFA and AMA), whereas samples with different feeding habits had a longer distance in the gut microbiota composition (Figure 5B). These results suggested that the dietary factors had a crucial influence on the gut microbiota composition.

### 3.4 The Impact of Dietary Habits on the Diversity and Composition of Gut Microbiota

**FIGURE 6:**
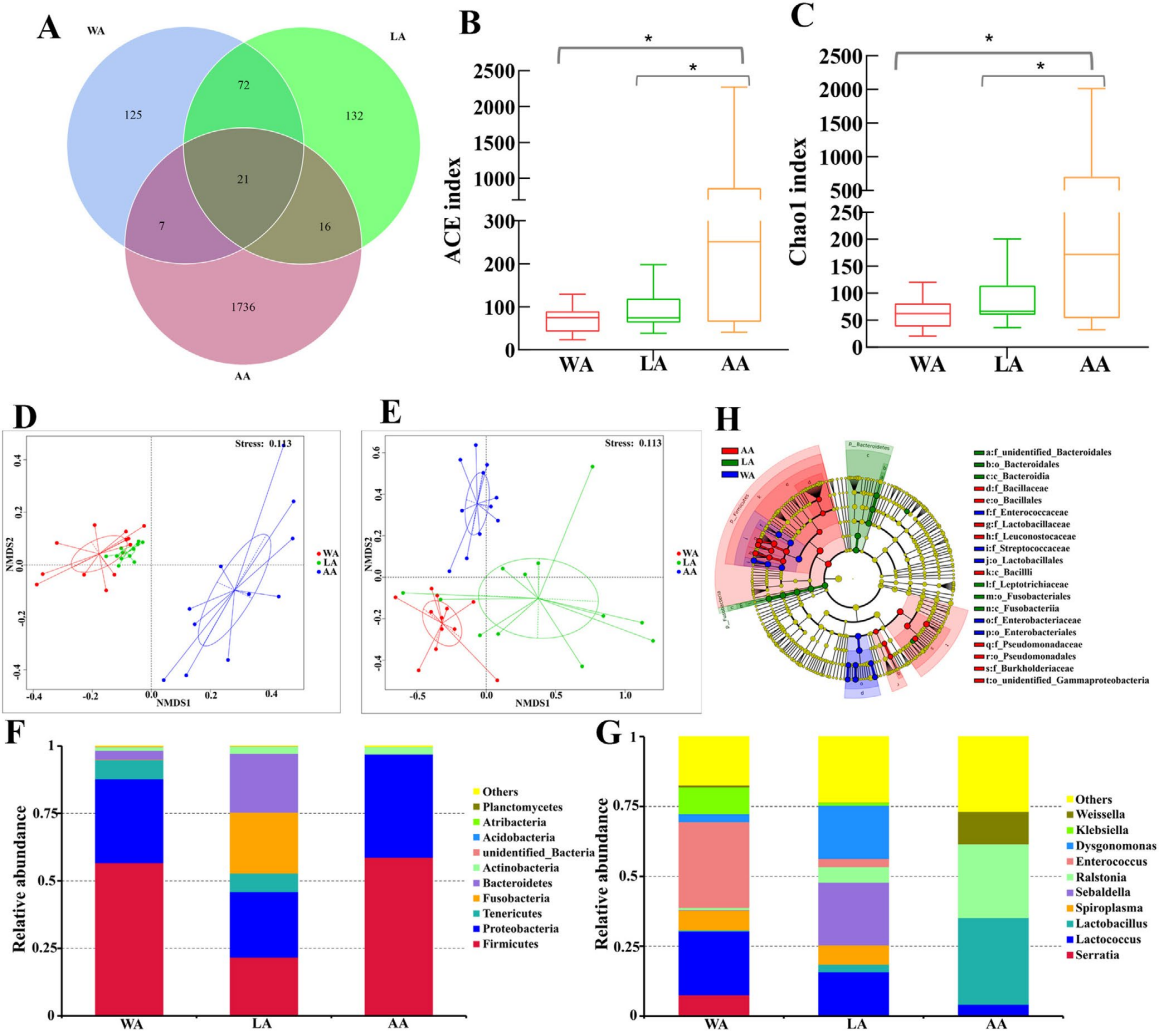
Analysis of gut microbiota in *Hycleus cichorii* with different diet habits. (A) Wayne plot of bacterial community at the OTU level; (B) ACE index, *X*‐axis: groups, *Y*‐axis: ACE index; (C) Chao1 index, *X*‐axis: Groups, *Y*‐axis: Chao1 index; (D) NMDS analysis based on unweighted UniFrac distance; (E) NMDS analysis based on weighted UniFrac distance; (F) Top 10 bacterial phylum with relative abundance, *X*‐axis: Groups, *Y*‐axis: Relative abundance of the bacteria; (G) Top 10 bacterial genera with relative abundance, *X*‐axis: Groups, *Y*‐axis: Relative abundance of the bacteria; (H) LEfSe analysis. The figure exhibited microbial communities with significant differences between the three groups (LDA > 4.0, *p* < 0.05). AA, adults fed with artificial diet; LA, adults fed with luffa flowers; WA, wild female adults. The asterisks denote statistically significant differences *p < 0.05.

According to different dietary habits, these samples were divided into three groups: WA (wild adult, *n* = 12), LA (luffa flower feeding group, *n* = 12), and AA (artificially fed group, *n* = 11). The gut microbiota of these three groups of samples was analyzed. At the OTU level, 21 OTUs were noted that were shared by the three groups of samples, which comprised 125 specific OTUs for WA, 132 specific OTUs for LA, and 1736 specific OTUs for AA (Figure 6A). No significant difference existed in ACE and Chao1 indices between WA and LA groups (*p* > 0.05). However, a significant difference was noted in ACE and Chao1 indices between the WA and AA groups, as well as between the LA and AA groups, indicating that the gut microbial diversity of AA group samples was significantly higher than that of the other two groups (Figure 6B,C). Beta diversity analysis was performed by unweighted UniFrac NMDS and weighted UniFrac NMDS for these three groups. The unweighted UniFrac NMDS exhibited a greater distance from the AA group and the other two groups (Figure 6D). In contrast, the weighted UniFrac NMDS revealed that the three sample groups clustered separately (Figure 6E). Hence, remarkable differences existed in the composition of gut microbiota among *H. cichorii Linnaeus* with different dietary habits, among which the gut microbiota of the AA group had a larger significant difference with respect to the other two groups. The microbial composition was analyzed at the phylum and genus levels with the three groups of samples. The top 10 bacterial phyla with respect to abundance were Firmicutes, Proteobacteria, Tenericals, Fusobacteria, Bacteroidetes, Actinobacteria, unidentified_Bacteria, Acidobacteria, Atribacteria, and Planctomycetes (Figure 6F). The top 10 abundant bacterial phyla were *Serratia*, *Lactococcus*, *Lactobacillus*, *Spirolasma*, *Sebaldella*, *Ralstonia*, *Enterococcus*, *Dysgonomonas*, *Klebsiella*, and *Weissella* (Figure 6G).

LEfSe analysis was conducted to analyze the differences in bacterial composition between different groups of samples (Figure 6H). The WA group did not have an iconic bacterial phylum. However, the LA group had Fusobacteria and Bacteroidetes as the iconic bacterial phylum. The AA group had Firmicutes as the iconic bacterial phylum (LDA > 4, *p* < 0.05). The iconic bacterial genera of the WA group were *Enterococcus*, *Lactococcus*, *Klebsiella*, *identified_ Enterobacteriaceae*, and *Citrobacter*. The LA group was marked by *Sebaldella*, *Dysgonomonas*, *Glutamiciber*, and *Comamonas*, whereas the AA group was marked by *Lactobacillus*, *Ralstonia*, *Weissella*, *Cupriavidus*, *Pedicoccus*, *Bacillus*, and *Pseudomonas* (LDA > 4, *p* < 0.05).

### 3.5 The Impact of Gender on the Diversity and Composition of Gut Microbiota

**FIGURE 7:**
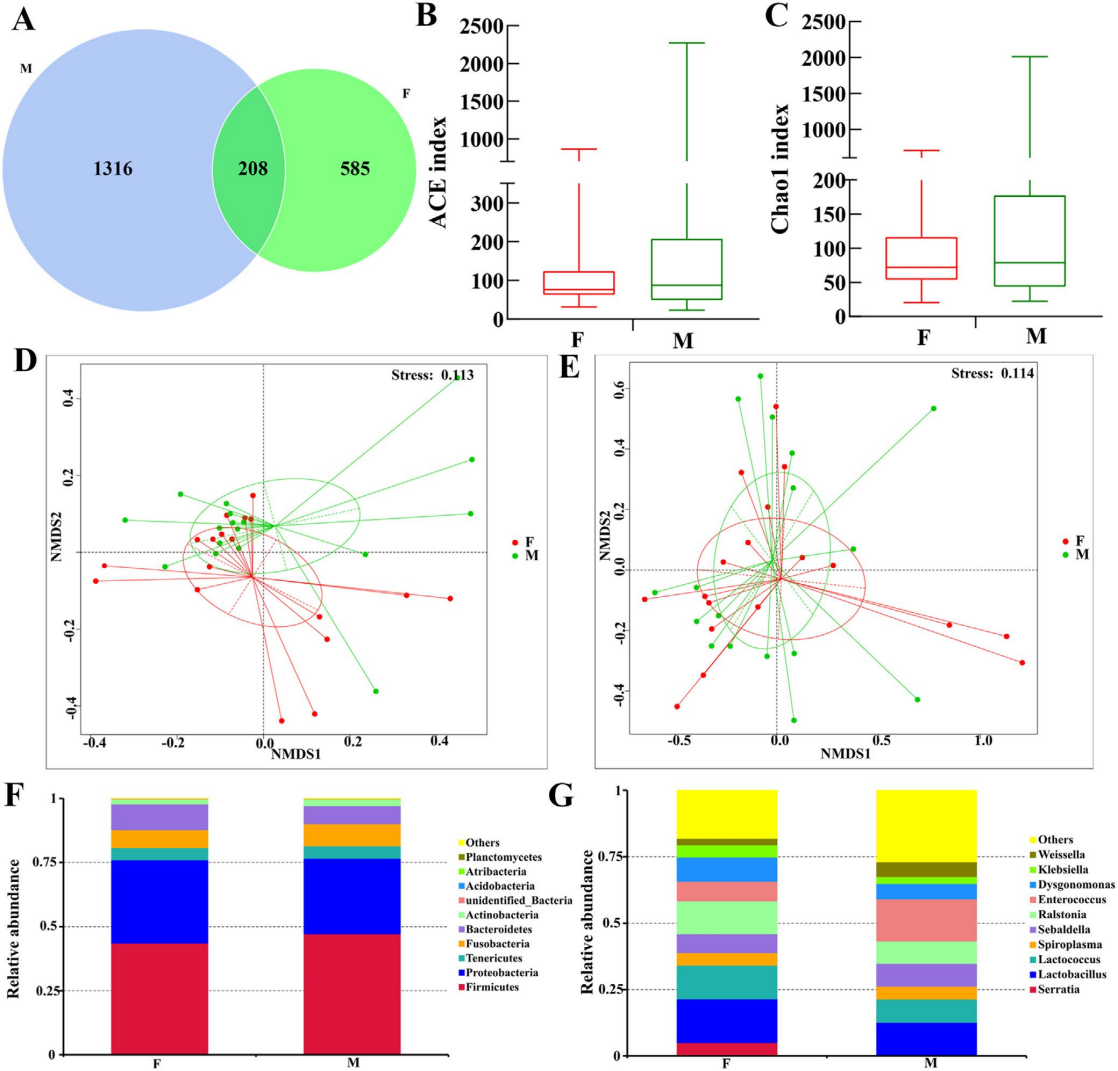
Analysis of the gut microbiota in *Hycleus cichorii* of different genders. (A) Wayne plot of bacterial community at the OTU level; (B) ACE index, *X*‐axis: Groups, *Y*‐axis: ACE index; (C) Chao1 index, *X*‐axis: Groups, *Y*‐axis: Chao1 index; (D) NMDS analysis based on unweighted UniFrac distance; (E) NMDS analysis based on weighted UniFrac distance; (F) Top 10 bacterial phyla with relative abundance, *X*‐axis: Groups, *Y*‐axis: Relative abundance of the bacteria; (G) Top 10 bacterial genera with relative abundance, *X*‐axis: Groups, *Y*‐axis: Relative abundance of the bacteria. F, female adults; M, male adults.

The samples were divided into two groups according to gender, F (female, *n* = 18) and M (male, *n* = 17), to analyze the gut microbiota in these two groups. At the OTU level, 208 OTUs were shared by the two groups of samples, with 1316 specific OTUs for the M group and 585 specific OTUs for the F group (Figure 7A). No significant difference existed in the diversity of ACE and Chao1 indices between the two groups (Figure 7B,C). Beta diversity analysis was performed by unweighted UniFrac NMDS and weighted UniFrac NMDS for these two groups. The absence of a significant clustering trend between the two groups of samples suggests that there were no significant differences in the gut microbiota of these groups (Figure 7D,E). The 10 most abundant bacterial phyla were identified as follows: Firmicutes, Proteobacteria, Tenericals, Fusobacteria, Bacteroidetes, Actinobacteria, unidentified_Bacteria, Acidobacteria, Atribacteria, and Planctomycetes (Figure 7F). The top 10 bacterial genera with respect to abundance were *Serratia*, *Lactococcus*, *Lactobacillus*, *Spirolasma*, *Sebaldella*, *Ralstonia*, *Enterococcus*, *Dysgonomonas*, *Klebsiella*, and *Weissella* (Figure 7G).

## 4 Discussion

Insect gut microbiota exhibit high diversity and group diversity at both intra‐ and inter‐species levels due to their diverse species, diverse ecological niches, wide insect feeding habits, and strong environmental adaptability (Pernice, Simpson, and Ponton 2014). It has a significant function in the life activities of insects, and researching gut microbiota is of great significance. In the present study, we detected the gut microbiota of *H. cichorii* adults, including 6 wild females (WFA) and 6 males (WMA), 6 luffa flower‐fed females (LFA) and 6 males (LMA), and 6 artificially fed females (AFA) and 5 males (AMA) using high‐throughput sequencing technology to determine the impact of dietary habits and gender on the composition and diversity of gut microbiota. The results suggested that the dominant bacteria in the gut of *H. cichorii* were Firmicutes (45.63%) and Proteobacteria (31.18%), similar to the dominant gut microbiota in most insects, such as *Apis cerana cerana* in the order of Hymenoptera (Zhang 2013), *Plutella xylostella* (Xia 2014), *Hepialus gonggaensis* (Liu et al. 2008), *Phalera assimilis* (Wen et al. 2015), *Phthorimaea operculella* (Zheng et al. 2017), and *Hyphantria cunea* (Wei et al. 2017) in the order of Lepidoptera. However, the gut‐dominant phyla of some insects comprised only Proteobacteria, such as *Cerambycidae*, *Ectropis oblique hypulina Wehrli*, *Ceratitis capitata* , and *Cnaphalocrocis medinalis* (Schloss et al. 2006; Behar, Yuval, and Jurkevitch 2008; Liu et al. 2016). The gut‐dominant phyla of other insects comprised only Firmicutes, such as *Melolontha hippocastanica Menetries* and *Clanis bilineata tingtauica* (Egert et al. 2005; Lyu and Liu 2009). Both Firmicutes and Proteobacteria were critical metabolic phyla in the gut, which can decompose fatty acids, complex sugars, and polysaccharides in food. This metabolic activity not only facilitates energy absorption for the hosts but also contributes to energy storage (Zhu et al. 2011; Samanta et al. 2012; Lu et al. 2012).

The comparison of differences showed that the diversity of gut microbiota of artificially fed *H. cichorii* was significantly higher than that of the lucerne‐fed and wild groups. Studies showed that the more diverse the food composition, the higher the diversity and richness of the host gut microbiota (Lau et al. 2018). Artificial fed *H. cichorii* had more abundant feed components, including soybean powder, maize pollen, and yeast powder, while the *H. cichorii* mainly feeds on bean flowers and loofah flowers in the wild. So the higher diversity of gut microbiota in artificial fed *H. cichorii* may be due to the more abundant food components.

Food and environmental factors exhibit a crucial impact on the composition of host gut microbiota (Amato et al. 2013; Barelli et al. 2015; Li et al. 2016; Studds et al. 2017). Zheng (2023) found that the dietary habits of insects in Ensifera were closely related to the composition of gut bacterial communities. Moreover, different species of insects with the same dietary habits had similar gut bacterial communities; however, there were significant differences in the composition and function of gut microbial communities among the same species of insects with different dietary habits. This finding is consistent with the results of the present study, which indicated that *H. cichorii* with different feeding habits had different compositions of gut bacteria.

The marker phylum of the AA group was Firmicutes, which was reported to degrade fibers and promote host absorption (Sun et al. 2023). The increase in the ratio of Firmicutes to Bacteroidetes (F/B ratio) was associated with obesity (Ley et al. 2006; Turnbaugh et al. 2009) and enhances the ability to obtain energy from the diet (Clarke et al. 2012). In the current study, the F/B ratio in the AA group was significantly higher than that in the WA and LA groups, so it was speculated that artificially fed *H. cichorii* may be better at efficiently utilizing food resources and providing energy for the body. The abundance of Fusobacteria and Bacteroidetes in the LA group was remarkably higher than that in the other two groups. Fusobacteria plays an important role in the production of butyrate, which, in turn, accelerates the accumulation of fat in the body and strengthens immunity (Panda et al. 2009). Bacteroidetes were commonly considered to be associated with chronic intentional inflammation (Parker et al. 2020). Hence, *H. cichorii* with different feeding habits regulated body metabolism via different types of gut microbiota. Therefore, food can drive the formation of gut microbiota.

Environmental factors exhibit a crucial impact on the composition of host gut microbiota. The iconic bacterial genera in the gut of *H. cichorii* in WA were *Enterococcu*, *Lactococcus*, *Klebsiella*, *identified_ Enterobacteriaceae*, and *Citrobacter*. The iconic bacterial genera in the gut of *H. cichorii* in LA were *Sebaldella*, *Dysgonomonas*, *Glutamicib, Comonas* and *Ralstonia*, *Weissella*, *Cupriavidus*, *Pediocus*, *Bacillus*, and *Pseudomonas* in AA. Except for *Lactococcus*, which was a probiotic (Zhao et al. 2023), other bacterial genera of the wild group (WA), such as *Enterococcus* (Osaili et al. 2018), *Klebsiella* (Wang et al. 2022), *unidentified bacteria_ Enterobacteriaceae* (Osaili et al. 2018), and *Citrobacter* (Lyu et al. 2023) are commonly found in soil environments. This may be due to the survival of the wild group of *H. cichorii* in the wild environment, where gut microbiota was influenced by the external environment and entered the body. The iconic bacterial genera in *H. cichorii* of LA were *Sebaldella* (Harmon‐Smith et al. 2010) and *Dysgonomonas* (Bridges and Gage 2020), which were both commonly found in insect intestines. *Glutamiciber* was widely present in water and soil environments (Wei et al. 2023), and *Comamonas* was a type of bacteria that can decompose pollutants effectively (Wang et al. 2020). In the gut of *H. cichorii* of the AA group, a large number of probiotic bacteria, such as *Lactobacillus*, *Weissella* (Liu et al. 2022), and *Pediococcus* (Porto et al. 2017), which were lactic acid bacteria and exerted beneficial effects on the intestines. Some common environmental bacteria, such as *Ralstoniais* (Peeters et al. 2013), *Cupriavidus* (Sohn et al. 2021), and *Pseudomonas* (Li et al. 2023) were also observed. These results indicated that environmental factors had a significant impact on host gut microbiota, and the artificial feed group *H. cichorii* had more beneficial gut microbiota. The possible reason was that the artificial feed had rich ingredients, which were beneficial for host development and metabolism.

Gender was also one of the factors affecting gut microbiota (Ding and Schloss 2014; Singh and Manning 2016). Lin et al. found that gender can significantly or extremely affect the microbiota in duck and chicken feces by 16S rRNA sequencing technology (Lin and Zhao 2020). In the present study, the diversity and composition of gut microbiota of *H. cichorii* with different genders were detected. The differential comparison showed that no remarkable difference existed in the diversity of gut microbiota between different gender groups. β diversity analysis indicated that no remarkable clustering trend was noticed between the two groups of samples. Hence, no remarkable difference was noticed in the composition of gut microbiota between these two groups of samples. This may be due to the inclusion of samples with different dietary habits in each group. Furthermore, the influence of dietary factors on gut microbiota is greater than that of gender. Therefore, in this study, gender did not significantly affect the gut microbiota diversity of *H. cichorii*.

However, this study had certain shortcomings. First, only 35 samples were selected, with only 5–6 samples detected in each group, which may not fully reflect *H. cichorii* gut microbiota diversity. Second, 16S rRNA high‐throughput sequencing technology also had certain limitations. 16S universal primers can lead to underestimation of specific populations, and OTU analysis can result in some sequences not being accurately annotated (Eisenstein 2018). In future research, we will consider expanding the sample size and using more precise metagenomic techniques to further analysis the intestinal microbiota in *H. cichorii*.

In conclusion, the present study analyzed the gut microbial diversity and composition of *H. cichorii* with different dietary habits and genders. The results showed that an elevated richness in food composition corresponds to a higher diversity of gut microbial species in the host. It suggests that food plays a significant role in the composition of the gut microbial community, which may consequently affect the nutritional metabolism and growth of the host. The study lays a basis for subsequent examination of gut microbiota in *H. cichorii* and other Coleoptera insects and provides insights into the artificial breeding of medicinal blister beetles.

## Author Contributions

**Chunyan Yi:** conceptualization (equal), data curation (equal), investigation (equal), methodology (equal). **Li Gao:** data curation (equal), investigation (equal), methodology (equal), writing – original draft (equal). **Cuicui Zhang:** resources (equal), visualization (equal). **Yanping Wang:** investigation (equal), methodology (equal). **Xu Liu:** formal analysis (equal), project administration (equal). **Yongli Yang:** resources (equal). **Song Chen:** formal analysis (equal), writing – review and editing (equal). **Chao Du:** conceptualization (equal), funding acquisition (equal), project administration (equal), writing – review and editing (equal).

## Conflicts of Interest

The authors declare no conflicts of interest.

## Data Availability

The datasets presented in this study can be found in online repositories. The names of the repository/repositories and accession number(s) can be found below: http://www.ncbi.nlm.nih.gov/bioproject/PRJNA1021773.
